# 192. Epidemiology, Microbiological Characteristics and Clinical Outcomes of Invasive Blood Stream Infections of Group B Streptococcal Isolates From Qatar

**DOI:** 10.1093/ofid/ofab466.394

**Published:** 2021-12-04

**Authors:** Maisa Ali, Mohammed Alamin, Gawahir Ali, Khalid Alzubaidi, Bashir Ali, Abdellatif Waqqad, Muna Almaslamani, Hamad Abdel Hadi

**Affiliations:** 1 HMC, doha, Ad Dawhah, Qatar; 2 Sidra, doha, Ad Dawhah, Qatar; 3 Communicable Disease Center, Doha, Ad Dawhah, Qatar

## Abstract

**Background:**

Group B Streptococci (GBS) or *Streptococcus agalactiae* colonize humans genitourinary and gastrointestinal tracts particularly of females. The pathogen is capable of causing invasive disease primarily in infants, pregnant and postpartum women as well as the elderly and patients with comorbidities. There is paucity of studies of the disease with regional differences in prevalence and presentation of invasive blood stream infection (BSI). In this study, we aim to assess prevalence, microbiological characteristics as well as clinical outcomes of invasive GBS disease from all ages groups at Hamad Medical Corporation (HMC), Qatar.

**Methods:**

A retrospective study was conducted on all patients with microbiologically confirmed GBS bacteraemia between January 2015–March 2019. Demographic, microbiological characteristics as well as clinical data were extracted from hospital information system.

**Results:**

Out of 196 confirmed cases of GBS blood stream infection, 63.7 % were females (125/196) of whom 44.8 % were pregnant (56/125), 53.6 % (30/56) were colonized while 36.3 % (71/196) were males. There were three distinct age group populations, paediatric less than 4 years of age at 35.7 %, young adults 25-34 (20.9 %) and the elderly > 65 year (17.4 %). Presenting symptoms were mild with fever recognised in only 53 % of cases (104/196) while 89% of cases had low Pitt bactermia score of 0-2. Microbiological characteristic using disc diffusion tests demonstrated all isolates were universally sensitive to penicillin (100%, 196/196) with significant resistance to clindamycin at 28.6 % (56/196) and erythromycin at 49 % (96/196) of which 34.4 % (33/96) had inducible clindamycin resistance. Clinical outcome showed high cure rate of 87.25% (171/196) with low complications at 8.76 % (17/196) and 4% (8/196) 30-day mortality.

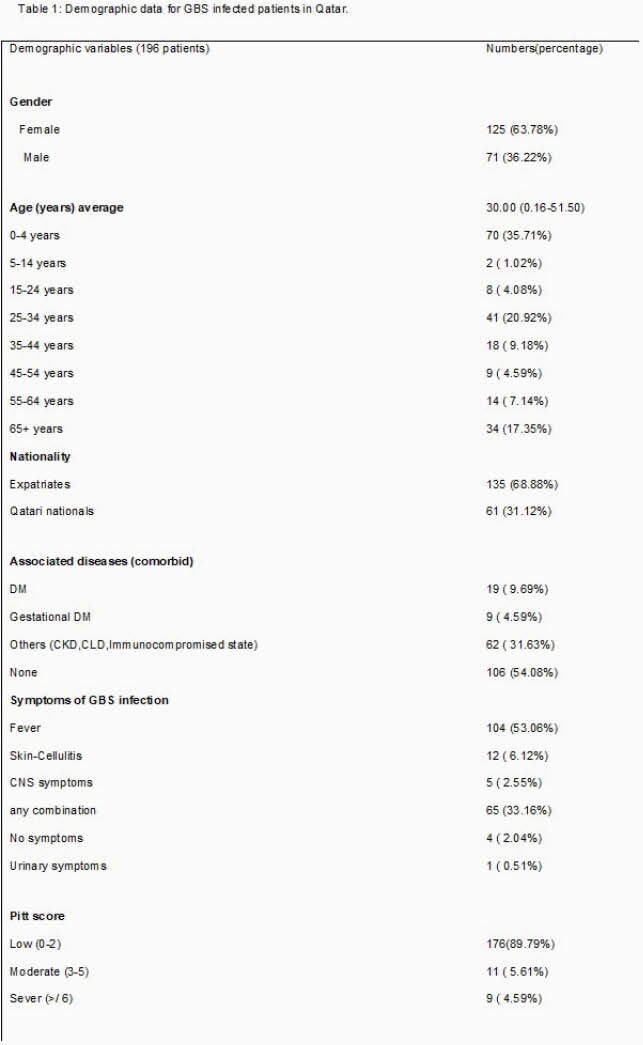

Antibiotic sensitivity profile for GBS isolates

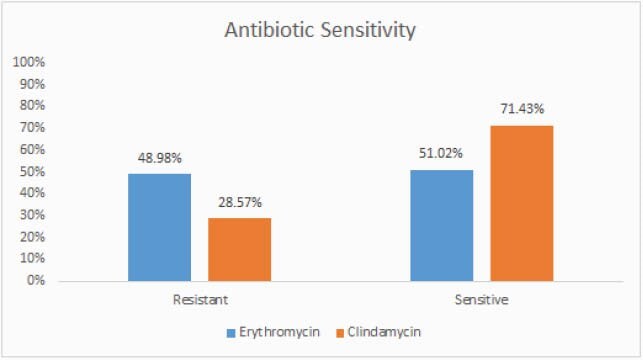

**Conclusion:**

*Streptococcus agalactiae* blood stream infection in Qatar is common in females, affects the very young, young adults and the elderly. Almost half of affected pregnant women are colonized. The organism remains universality sensitive to pencilling with significant resistance to clindamycin and erythromycin. Patients presents with mild symptoms with high cure rates, low complications and safe outcome for the majority of cases.

**Disclosures:**

**All Authors**: No reported disclosures

